# Documenting Paintings with Gigapixel Photography

**DOI:** 10.3390/jimaging7080156

**Published:** 2021-08-21

**Authors:** Pedro M. Cabezos-Bernal, Pablo Rodriguez-Navarro, Teresa Gil-Piqueras

**Affiliations:** 1 Departamento de Expresión Gráfica Arquitectónica, Universitat Politècnica de València, 46022 Valencia, Spain; 2 Centro de Investigación en Arquitectura, Patrimonio y Gestión para el Desarrollo Sostenible (PEGASO), Universitat Politècnica de València, 46022 Valencia, Spain; rodriguez@upv.es (P.R.-N.); tgil@ega.upv.es (T.G.-P.)

**Keywords:** gigapixel photography, ultra-high resolution, art documentation, stitching, image registration, virtual musealization

## Abstract

Digital photographic capture of pictorial artworks with gigapixel resolution (around 1000 megapixels or greater) is a novel technique that is beginning to be used by some important international museums as a means of documentation, analysis, and dissemination of their masterpieces. This line of research is extremely interesting, not only for art curators and scholars but also for the general public. The results can be disseminated through online virtual museum displays, offering a detailed interactive visualization. These virtual visualizations allow the viewer to delve into the artwork in such a way that it is possible to zoom in and observe those details, which would be negligible to the naked eye in a real visit. Therefore, this kind of virtual visualization using gigapixel images has become an essential tool to enhance cultural heritage and to make it accessible to everyone. Since today’s professional digital cameras provide images of around 40 megapixels, obtaining gigapixel images requires some special capture and editing techniques. This article describes a series of photographic methodologies and equipment, developed by the team of researchers, that have been put into practice to achieve a very high level of detail and chromatic fidelity, in the documentation and dissemination of pictorial artworks. The result of this research work consisted in the gigapixel documentation of several masterpieces of the Museo de Bellas Artes of Valencia, one of the main art galleries in Spain. The results will be disseminated through the Internet, as will be shown with some examples.

## 1. Introduction

New techniques for the dissemination of cultural heritage through digital media is one of the lines of research providing benefits to society since the research results can be available to the public by means online virtual museum displays. In fact, many important international museums [1], are using these technologies in collaboration with the multinational Google, which has developed its own high-resolution digital capture system for paintings. These works are exhibited on the Arts and Culture Project website [2], which contains many artworks that can be displayed with a high level of detail. They are digital reproductions with a gigapixel resolution, that is, a resolution greater than 1000 megapixels, which is 50 times greater than the image resolution provided by a conventional digital camera.

**Figure 1:**
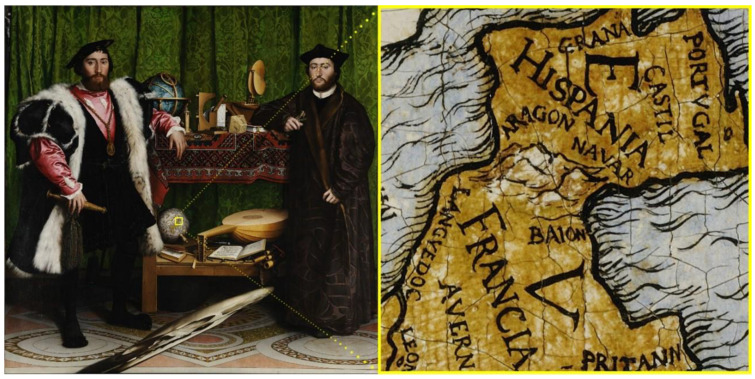
Hans Holbein the Younger, 1533, *The Ambasssadors*. Picture and detail obtained from Google Arts and Culture [2].

Gigapixel images allow documenting and analysing the paintings accurately, which is very useful for curators and art scholars. Furthermore, the virtual visualizations that can be generated from this type of image make the artwork accessible to anyone connected to the Internet. The viewers will be able to dive into the work, in such a way, that they would appreciate many details, which would be negligible to the eye in a real visit (Figure 1).

Apart from Google, there are very few companies specialized in capturing gigapixel images of artworks due to the technical complexity and the specialized equipment. There are some examples, such as the French state organization *Centre de Recherche et de Restauration des Musées de France* CR2MF [3], the Italian company *Haltadefinizione* [4], or the Spanish Madpixel [5]. Some researchers have used gigapixel photography for documenting rupestrian paintings [6] and even combined with multispectral imaging techniques [7]. Other research works have used gigapixel images for documenting large Baroque illusionistic paintings [8,9].

Digital photographic capture with gigapixel resolution is not an easy task and serious difficulties may arise due to physical problems such as the diffraction of light, which represents a barrier that limits the sharpness that can be obtained with an optical device and a digital sensor. For this reason, the progression in the resolution of digital sensors has already reached the limit established by the diffraction of light and by the optical resolution provided by the lenses [10] that cannot match the resolution of the current top digital sensors. Going beyond this would not take advantage of the effective resolution of the sensor, unless the size of the optical-sensor assembly were increased, which is not practical for the future development of conventional cameras. In fact, there have been few attempts to develop prototype cameras providing gigapixel images on the fly. One of the first was the large format camera developed within the *Gigapxl Project* by Graham Flint in 2000 [11]. In this bulky camera, the image was exposed on a 450 × 225 mm negative film, which was later digitized to form a 4 gigapixel (Gp) image. Another more recent proposal is the AWARE 2 gigapixel camera, started by D. Brady and his team in 2012. This prototype included 98 micro-cameras with a combined resolution of 1 Gp and it is still being developed and improved. The AWARE 10 and 40 models reach almost 3 Gp. Nevertheless, such special prototypes are far from becoming an accessible option due to their high complexity and cost.

A reasonable solution to overcome the problem of diffraction and achieve gigapixel images using conventional cameras is multi-shot panoramic capture, which consists in obtaining a set of photographs from the same point of view and with a sufficient overlap between adjacent pictures so that, by means of image stitching software, they can be joined to compose a higher resolution image [12].

**Figure 2:**
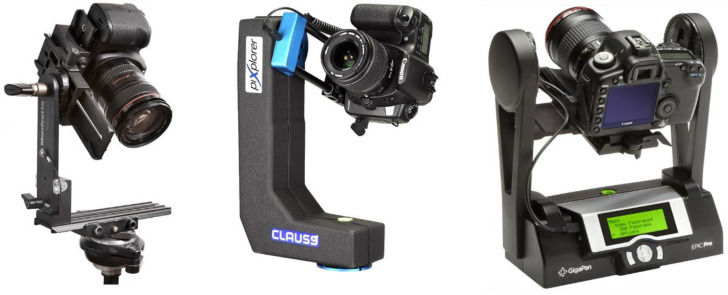
Manual and motorized panoramic heads.

In order to achieve a perfect stitch between pictures and to avoid parallax errors, it is mandatory to use a panoramic head (Figure 2) to fix the position of the optical centre or no-parallax point of the lens, while rotating the camera to obtain the different shots that will compose the final image. Moreover, a telephoto lens should be used so as to maximize the final resolution.

The capture system developed by Google, called Art Camera [13], seem to be based on this same principle, and consists of a camera integrated in a panoramic head that progressively sweeps the painting from a fixed point. The partial images are subsequently processed by the multinational itself. This type of camera is not for sale and there are only a very limited number of units available exclusively to the company.

The capture systems used by other commercial companies such as Haltadefinizione, are not fully described, but some of them are based on using high-end digital reflex cameras and high-cost automated panoramic heads by Clauss [14]. Madpixel has his own automatic panoramic head called MadpixelROB [15]. The aforementioned research works have also used the panoramic head methodology.

Using a panoramic head and a telephoto lens is a very effective method for documenting relatively small paintings with gigapixel resolution, but when it comes to capturing artworks of moderate size, there arise some drawbacks that limit image sharpness due to narrow depth of field provided by long focal length lenses. This problem will be discussed and fixed by following the techniques that will be revealed below, since one of the main aims of this article is to show a new and very accurate gigapixel capture methodology that uses relatively affordable equipment. Moreover, the digital image processing to generate the gigapixel image and the dissemination of the results on the Internet will be explained and carried out by means of opensource software.

## 2. Materials and Methods

### 2.1. Capture Techniques

The photographic equipment that was used in this research work consisted of a 32.5 Mp digital camera Canon EOS 90D, equipped with a fixed telephoto lens Canon EF 200 mm f2.8 L II USM, a Manfrotto tripod 28B and a Manfrotto panoramic head 303 SPH.

Long focal length lenses should be used to maximize resolution and the camera must be quite close to the artwork, so that, in these circumstances, depth of field provided by the lens is very short. Furthermore, this is aggravated by the need to use an intermediate diaphragm aperture to avoid losing global sharpness by the effect of light diffraction [16]. This problem causes a loss of sharpness in certain zones of the off-centred shots of the paintings, when using the panoramic head capture methodology. This is due to the angular deviation between the camera sensor and the painting.

**Figure 3:**
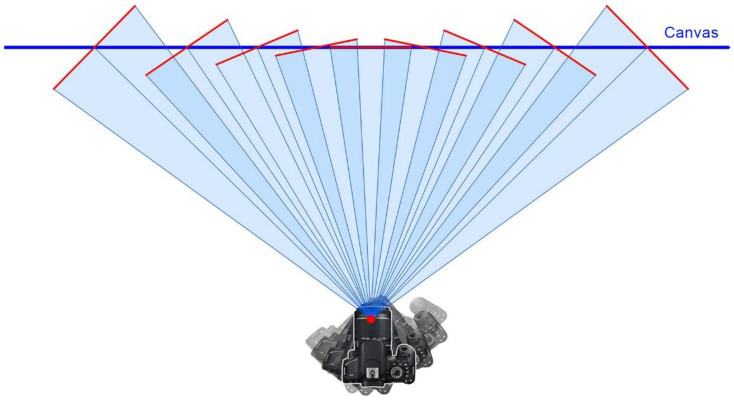
Obliquity between the shots and the canvas when using a panoramic head.

**Figure 4:**
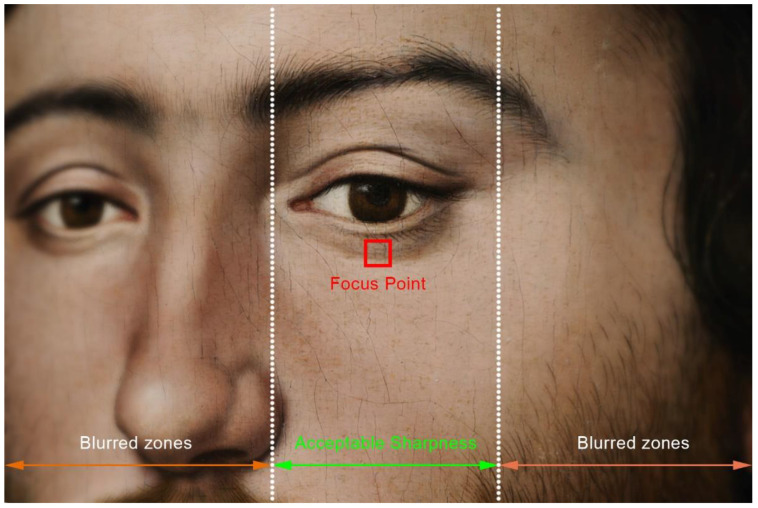
Loss of sharpness in an oblique shot due to narrow depth of field of long focal lenses.

When the digital sensor remains parallel to the painting, all the points are perfectly focused, so the distance between them and the sensor remain the same. However, as the camera is rotated towards the picture margins, the angle between the sensor and the painting increases dramatically (Figure 3). This causes only those points that are at the same distance from the camera sensor as the focused point to be perfectly sharp, while those remaining at different distances, will be gradually blurred due to narrow depth of field (Figure 4).

**Figure 5:**
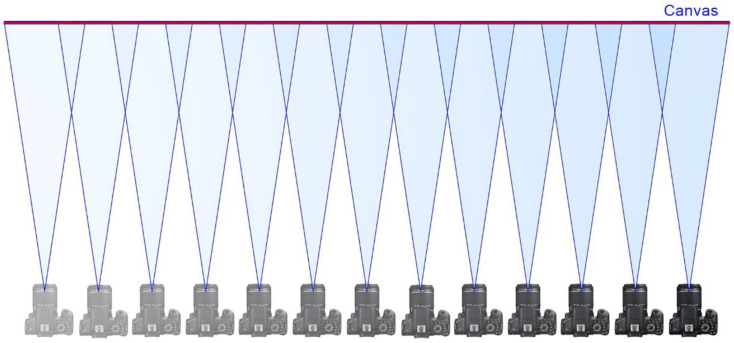
Parallel camera translation to take all the pictures frontally to the canvas.

To solve this problem, a new capture technique has been put into practice, which consists in taking the shots while moving the camera parallel to the painting (Figure 5). Doing so, the sharpness of the pictures will be always optimal and never limited by depth of field. However, while this method would solve the problem of sharpness, it also generates some difficulties that must be overcome. On the one hand, as the viewpoint would vary continuously, the light reflected by the work will change between the shots, causing slight differences in exposure and problems with specular reflections. Those issues can be solved by using a strategically placed controlled light source, which moves along with the camera.

Another problem arises when it comes to joining the photo mosaic, obtained in that way, so most of the current software for stitching images would be unable to join the mosaic when the viewpoint is not static. Fortunately, this problem can be solved with the help of some stitching algorithms, initially developed by the German professor Helmut Dersch [17], which were implemented in Hugin [18], an opensource stitching software with general public license that allows joining pictures taken from different viewpoints, as long as they represent a planar surface. Optionally, the commercial software PTGUI [19] would also do an excellent job when stitching these kinds of photomosaics.

Another way to construct the gigapixel image is by means SfM (structure from motion) photogrammetry software, since the construction algorithm is conceived to generate a textured mesh from pictures taken from different viewpoints. The problem is that it would be needed a greater number of shots since a minimum overlap of 50% between adjacent pictures is advisable, while a 30% overlap would be fine when using stitching software. However, an advantage of using SfM photogrammetry software would be the 3D reconstruction of the painting and even the frame, which is not a plane surface, while stitching software would provide an accurate 2D rectified orthophoto of the canvas but not of the volumetric frame.

**Figure 6:**
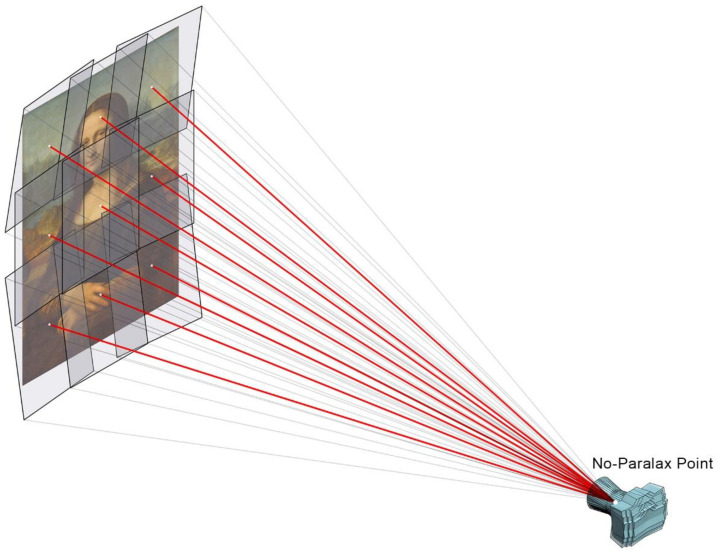
Single-viewpoint capture technique. The camera is rotated around its no-parallax point.

Taking into account the previous considerations, the following gigapixel capture techniques are proposed, which will be suitable for paintings or planar surfaces:

The single-viewpoint capture technique is a known and common methodology, which consist in using a panoramic head to rotate the camera around the no-parallax point, which would be fixed and centred in front of the canvas, thus obtaining a set of overlapping pictures (Figure 6). This technique requires limiting the obliquity of the shots in relation to the canvas in order to preserve the maximum image sharpness.

**Figure 7:**
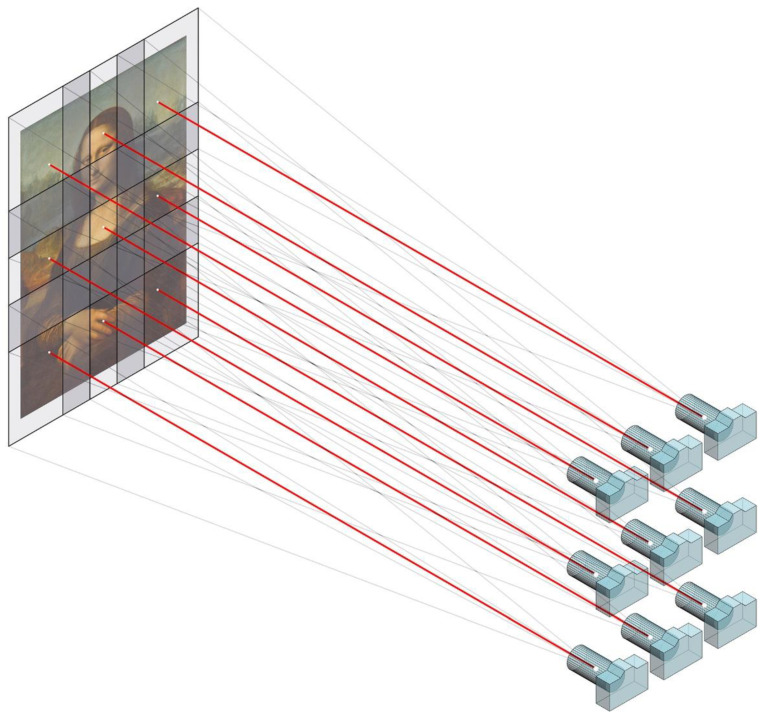
Parallel-multi-viewpoint capture technique. The camera moves parallel to the canvas.

The parallel-multi-viewpoint capture technique is a new method, which consist in taking a mosaic of overlapped pictures as the camera is moved in parallel to the canvas describing several rows or columns. The camera digital sensor must be parallel to the artwork, or, in other words, the lens optical axis should be orthogonal to the canvas (Figure 7).

**Figure 8:**
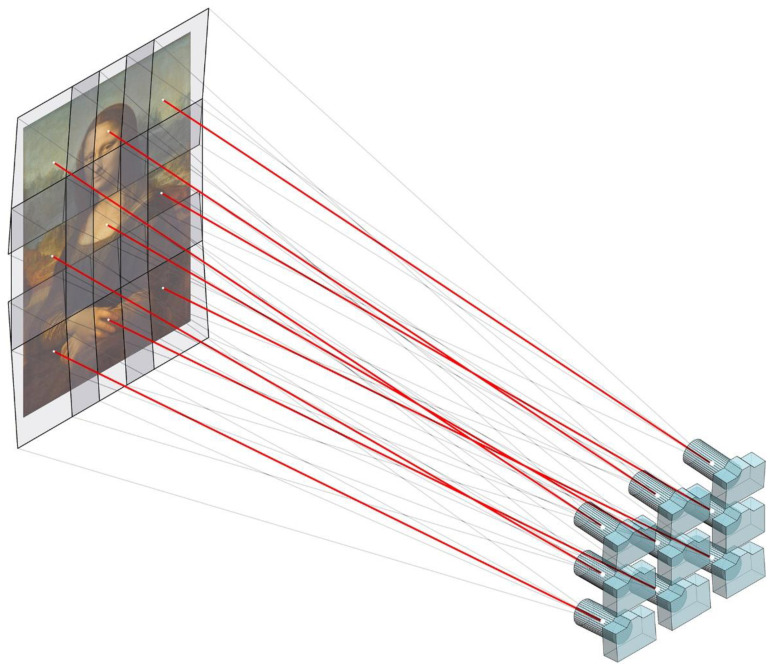
Tilted-multi-viewpoint capture technique. The camera is moved mostly parallel to the canvas and can be tilted if needed. Normally, only the upper or lower rows would be tilted.

The tilted-multi-viewpoint capture technique is a variation of the previous one, in which the camera can be tilted if required. This option may seem awkward but can be useful in the case of capturing large paintings with a tripod that is not tall or short enough to allow the parallel movement of the camera in front of the whole painting. In that case, the camera could be tilted when taking the upper or lower rows to cover the entire canvas. (Figure 8).

The resolution of the final gigapixel image, achieved with those methods, will depend on several factors, such as the camera sensor resolution, the lens focal length, and the distance between the canvas and the camera, as will be discussed later.

### 2.2. Illumination Set

When capturing paintings there are several approaches to illuminate the artwork properly. The simplest way is to use the museum’s own illumination when the capture process is carried out in situ. Nevertheless, for a uniform and colour-accurate result it would be advisable to use dedicated and controlled light sources. In this research, there were used two Mettle 28 inch soft boxes lights, providing a colour temperature of 5500 K and a CRI (Colour Rendering Index) greater than 90.

**Figure 9:**
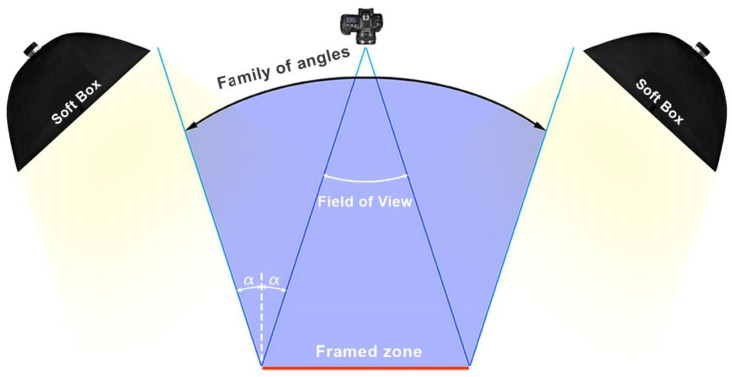
Lighting scheme to illuminate the artwork uniformly. In order to avoid specular reflections on the canvas, the light sources must be placed outside of the volume defined by the family of angles.

The soft boxes must be placed strategically to avoid specular reflections on the canvas and to guarantee lighting uniformity, so the lighting scheme proposed in Figure 9 is advisable. The lights must be placed outside the family of angles zone, as otherwise specular reflections may occur.

**Figure 10:**
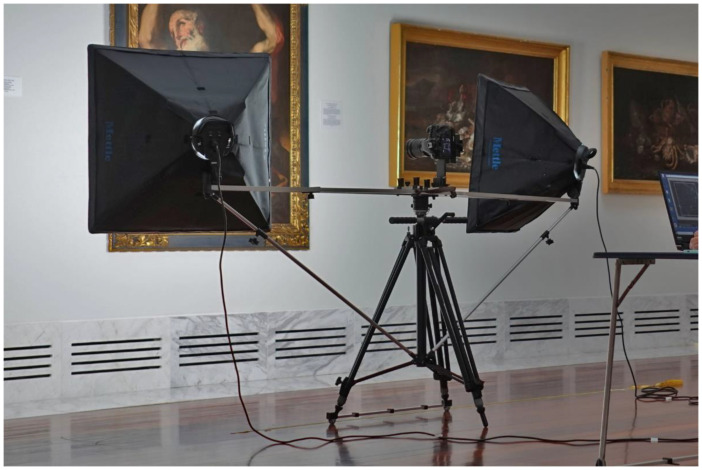
Lighting mount to attach the lighting system to the tripod.

When using the multi-viewpoint capture technique, it is mandatory to move the lighting system along with the camera, so a special mount was designed to attach the soft boxes to the tripod (Figure 10). Additionally, a sliding system for the tripod’s feet, which consisted in a two-wheeled aluminium bar, was also constructed to facilitate the movement of the set.

### 2.3. Camera Settings and Colour Accuracy

It is advisable to use an intermediate diaphragm aperture value to avoid the diffraction phenomenon while maintaining good depth of field. Setting the camera to f8 would be advisable as it is usually the golden number for maximizing image sharpness. The camera speed will depend on the lighting conditions. Slow speeds will not be a problem since a tripod is used.

The camera ISO value must be at the lower setting to avoid noisy pictures. An ISO 100 value is advisable. RAW Image file format should be used instead of JPG in other to maximize the dynamic range and to properly adjust the white balance and to apply an accurate colour profile during the digital developing process.

When using telephoto lenses, it is advisable to shoot the camera remotely, even when a tripod is used. For this reason, it is recommended using a remote shooter or, even better, a mobile device or notepad with a compatible application for shooting the camera. In this research, a notebook with the free software EOS Utility by Canon, was used to control the camera via Wi-Fi. 

**Figure 11:**
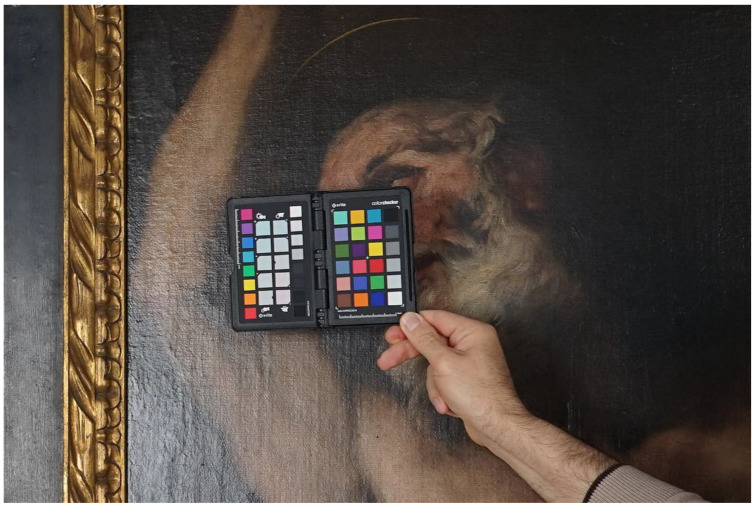
Reference picture with the X-Rite ColorChecker chart that will help to set the proper white balance and to create a specific colour profile for the lighting conditions.

In order to capture the colours of the artwork accurately, a Xrite ColorChecker chart was used to create a colour profile from a picture of the chart, which was illuminated by the scene lights (Figure 11). The colour profile was generated by the ColorChecker Camera Calibration software by Xrite and it can be used with RAW developers, such as the opensource RawTherapee [20], as well as with other commercial software, such as Adobe Camera Raw or Adobe Lightroom [21]. The picture of the chart can also be used as a reference to adjust the proper white balance of all the shots at once, by using the middle grey patch of the chart to neutralize any colour predominance.

### 2.4. Generating the Gigapixel Image

In order to join the set of pictures obtained with any of the aforementioned techniques, a stitching software such as Hugin or PTGUI can be used. As mentioned before, Hugin is a powerful opensource stitching software that can even calculate the camera translation between shots, when the multi-viewpoint technique is used. The only drawback of Hugin is that is slow when compared to the commercial alternative PTGUI, which is incredibly fast.

The workflow with the stitching software is very simple. Initially, the set of pictures are analysed to automatically detect homologous points in the overlapping zones of the shots. Then, the optimizer algorithm uses all these points to calculate the spatial position of every picture, which is defined by the pitch, yaw, and roll angles. In addition to this, the camera translations, and the parameters to correct the radial distortion, caused by the lens, are also computed. The final step allows rendering the set of pictures into a whole image at gigapixel resolution. The maximum final resolution will depend on the number of shots and their original resolution. That is estimated by the software so as to avoid incremental interpolations.

When using the multi-viewpoint technique, it is possible to assemble the gigapixel image with an automated SfM photogrammetry software, such as Agisoft Metashape. As mentioned before, using this kind of software would require a greater overlapping between adjacent shots and it is much more time consuming. This option would be interesting only when documenting volumetric artworks, such as altar pieces. The workflow for this kind of software begins with the determination of the camera positions from the analysis of the homologous points and the sparse point cloud. Then, the dense cloud of the model can be computed, and can be later triangulated to form a polygonal mesh. This mesh can be texturized by projecting the original pictures onto the mesh. Finally, an orthophoto of the 3D textured model can be generated at gigapixel resolution.

It should be mentioned that SfM photogrammetric techniques would not provide an absolutely accurate three-dimensional restitution of those subtle surface details, but since our main objective is obtaining an orthophoto of the painting, it would provide excellent results. In order to render fine surface properties of the painting, reflectance transformation imaging (RTI) would be very useful, as well as other much more expensive 3D scanning techniques [22].

### 2.5. Canvas Measurement

Knowing the real dimension of the paintings is important to provide an accurate scale of the gigapixel image. Normally the dimensions of the artwork are specified on the wall labels, but they are approximated. A Topcon Image IS robotic total station was used for taking the measurement of the canvases without touching the artwork. This total station has a reflector-less measurement capability between 1.5 and 250 m, providing a short-range precision of ±5 mm (MSE), and an angular precision of 0.3 mgon. The instrument was placed in front of the painting, and, with the help of the telescopic sight, the corners of the canvas were identified. The coordinates of these points were obtained using TopSurv, a software that comes with the total station.

## 3. Results

In this section, some examples, which were captured using the described techniques, will be exposed. All the paintings belong to the Museo de Bellas Artes of Valencia (Spain).

### 3.1. Single-Viewpoint Capture

**Figure 12:**
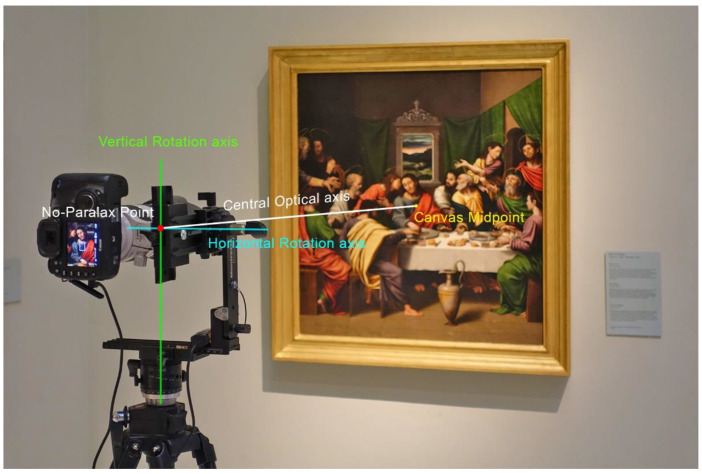
Single-viewpoint capture technique. Viewpoint position in the capture of the *Santa Cena* by Juan de Juanes (1534). *Museo de Bellas Artes de Valencia* (Spain).

The first example is focused on the artwork entitled *La Santa Cena*, painted by Juan de Juanes in 1534. This artwork measures 92.3 cm tall × 85.8 cm wide. Due to its reduced size, it was shot using the Single-Viewpoint Technique. The camera was placed in a central position with respect to the canvas and at a distance of 2 m. In this manner, the perimeter parts were not too oblique to the camera sensor, thus preserving the image sharpness (Figure 12). All the zones had to be inside depth of field limits provided by the telephoto lens. The diaphragm was set to f8, the ISO value to 100, and the shutter speed was set to 2 s in accordance with the lighting conditions.

**Figure 13:**
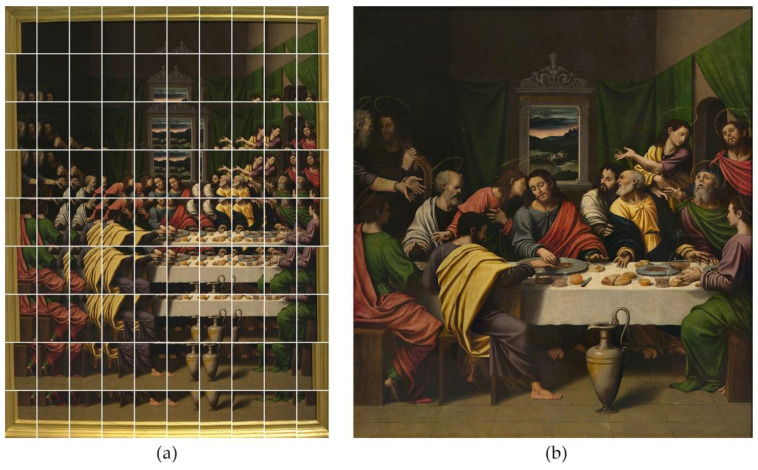
(**a**) Set of 90 pictures taken during the gigapixel capture. (**b**) Stitched gigapixel Image.

A total of 90 photographs were taken by rotating the camera around its centre of perspective, in steps of 2.5 degrees (horizontal rotation), and 4 degrees (vertical rotation). That resulted in a mosaic of 10 columns and 9 rows, with an overlapping between adjacent pictures greater than 30% (Figure 13a).

A reference picture, with the X-Rite Colour Checker Chart, was taken to generate a specific colour profile for the lighting conditions of the scene, and to adjust the white balance in the RAW development process precisely. In this case, only the lights of the museum were used as the lighting source.

The development process was carried out using the opensource software RAW Therapy. There were generated 90 files in TIF format with 32 bits per channel, which were perfectly balanced with the colour profile created by the ColorChecker Camera Calibration software, which is provided by the manufacturer of the colour chart.

After the raw development process, the mosaic was joined using Hugin, a GPL-licensed stitching software, which generated the final image (Figure 13b), with a resolution of 26,511 × 28,520 px, that is, 756 megapixels (0.76 gigapixels).

It is interesting to express the resolution relative to a measurement unit, so this value would be independent of the artwork size. This parameter is known as pixel density and will allow an objective comparison of the level of detail, acquired in each case. The pixel density, expressed in pixel per inch, was 787 ppi in this case.

**Figure 14:**
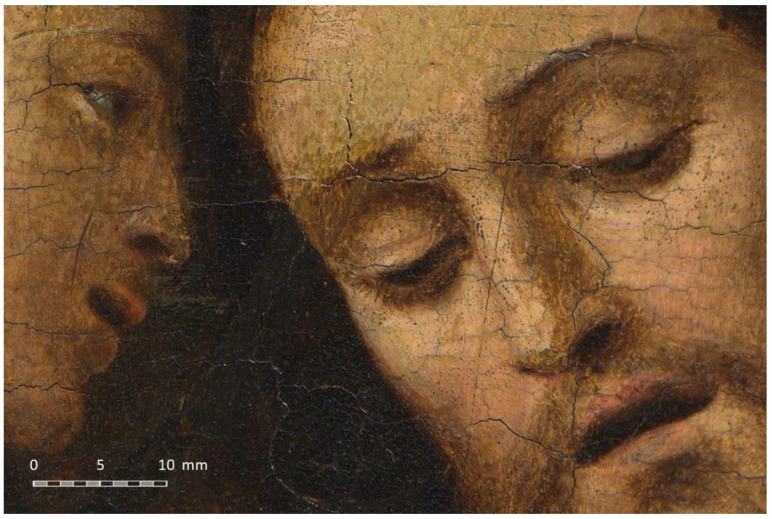
Fragment of the resulting gigapixel image.

Figure 14 shows a fragment of the resulting image in which the level of detail can be noticed. The pixel density is not huge because of the long distance between the camera and the canvas. This distance was increased to prevent the loss of sharpness in the decentred shots due to the obliquity between the canvas and the camera sensor. Nevertheless, a greater resolution could have been achieved if the proposed parallel multi-viewpoint capture method had been used, so the camera would have been placed almost at the minimum focusing distance of the lens (1.5 m instead of 2 m).

### 3.2. Parallel Multi-Viewpoint Capture

The parallel multi-viewpoint capture method will produce better image quality than the previous one since the camera sensor is always parallel to the canvas. Consequently, all the zones of the pictures would be perfectly focused. Nevertheless, this approach is more complicated, so the camera must be moved along with the lights every shot, but it is worth it.

This technique was put into practice in the next example to capture the painting entitled *Virgen de la Leche*, painted by Bartolomé Bermejo around 1478. The canvas size is quite small, measuring 49.1 cm tall × 34.5 cm wide. 

In this case, the previously described lighting system was used, which consisted of two soft boxed attached to the tripod (Figure 10). The lamps of the museum were turned-off so as not to mix different light sources.

**Figure 15:**
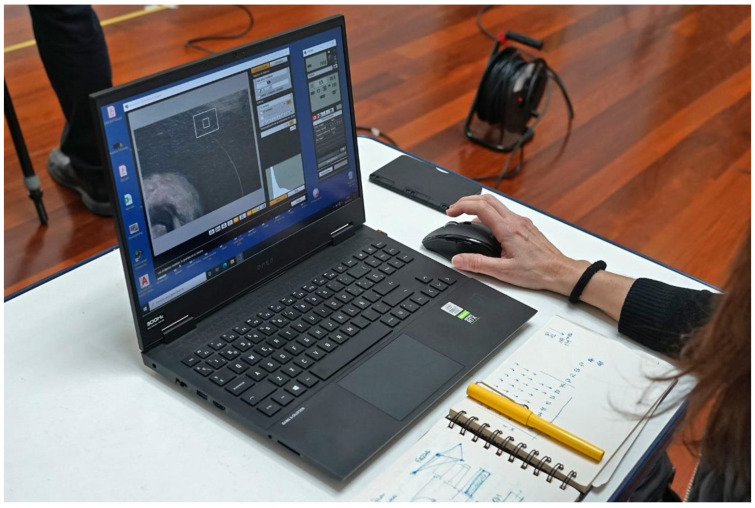
Remote control of the camera with the laptop via Wi-Fi.

The photographic set must be stabilized after changing the camera position, so the flexibility of the light stand, which is attached to the tripod, can lead to slightly blurred shots due to small vibrations transmitted to the camera. This is controlled by monitoring the camera with the laptop. The live view image, which is received via Wi-Fi from the camera, is magnified 10× to detect any slight vibration and to focus the canvas very accurately. Once the image is completely steady and focused, the camera is remotely shot (Figure 15).

**Figure 16:**
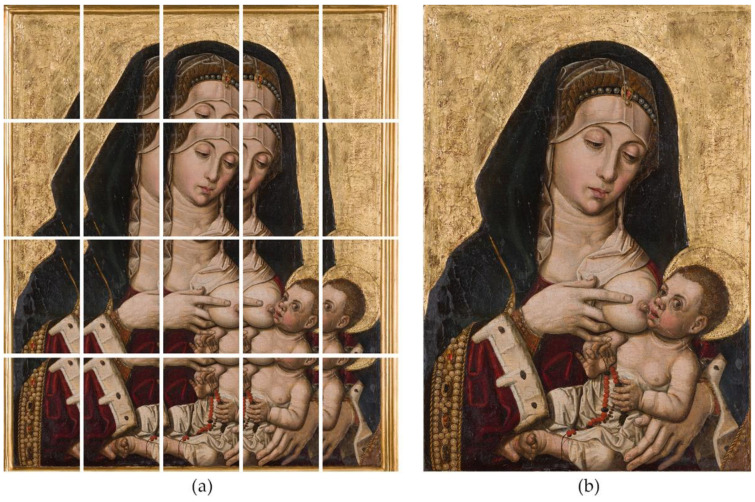
(**a**) Set of 20 pictures taken during the gigapixel capture. (**b**) Stitched Gigapixel Image.

We took 20 shots to cover the entire canvas obtaining a mosaic of 5 columns and 4 rows with an overlapping greater than 30% (Figure 16a). The camera was placed at 1.5 m from the canvas and moved horizontally to cover the upper row. Then, the camera was moved downwards vertically to initiate the second row and so on. A measuring tape was attached to the floor, parallel to the canvas, to serve as a reference for the translation movement. The diaphragm was set to f8, the ISO value to 100, and the shutter speed was set to 1/20 of second according to the lighting conditions.

**Figure 17:**
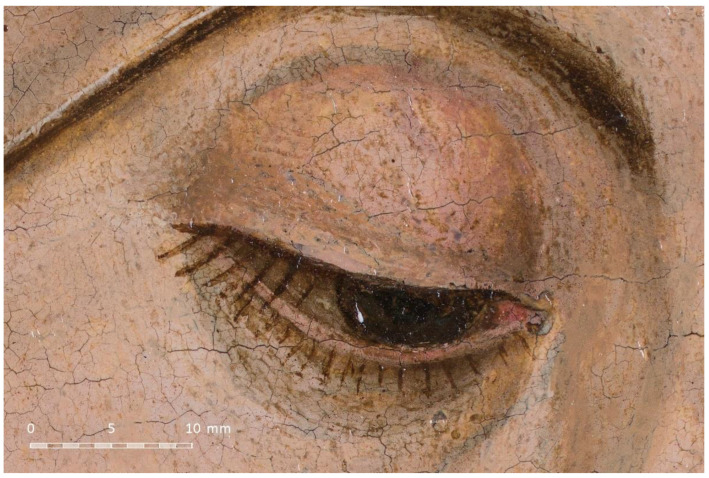
Fragment of the resulting gigapixel image.

The raw developing process was carried out in the same way as the previous example. Nevertheless, the shots were stitched using PTGUI instead of Hugin as it is much faster. The resulting image (Figure 16b) has a final resolution of 14,183 × 20,199 px, which provides a pixel density of 1044 ppi. This is much better than in the previous case, since the camera could be placed closer to the canvas. As mentioned, there is no problem with depth of field when using this method. Figure 17 shows a cropped fragment of the gigapixel image centred on the eye of the Virgin.

### 3.3. Tilted Multi-Viewpoint Capture

The last example shows the gigapixel capture of the artwork entitled Martirio de San Bartolomé, painted by Luca Giordano around 1650. The canvas size is 136 cm tall × 95 cm wide, which is a bit large to use the single-viewpoint method properly, so image sharpness would be poor in some zones of the boundary shots. Moreover, the upper and lower borders of the painting exceeded the maximum and minimum height of the tripod, so the tilted multi-viewpoint capture method was chosen in this case.

**Figure 18:**
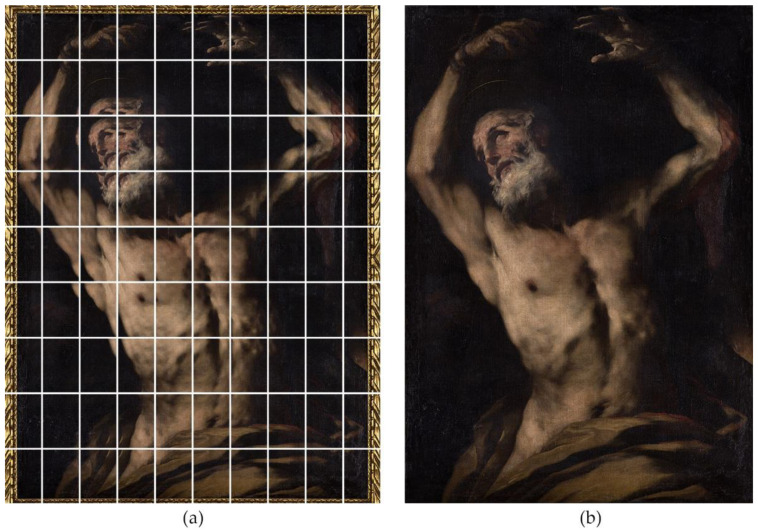
(**a**) Set of 90 pictures taken during the gigapixel capture. (**b**) Stitched gigapixel image.

We took 90 shots, forming a mosaic of 10 columns and 9 rows (Figure 18a). Most of the pictures where parallel to the canvas. Only the shots corresponding to the upper and lower rows were slightly tilted vertically to cover the entire artwork, which is negligible for the image quality. The distance from the camera to the canvas was set to 1.9 m. The camera settings were: diaphragm f8, ISO 100, and camera speed 1/5 of second, accordingly to the lighting conditions.

**Figure 19:**
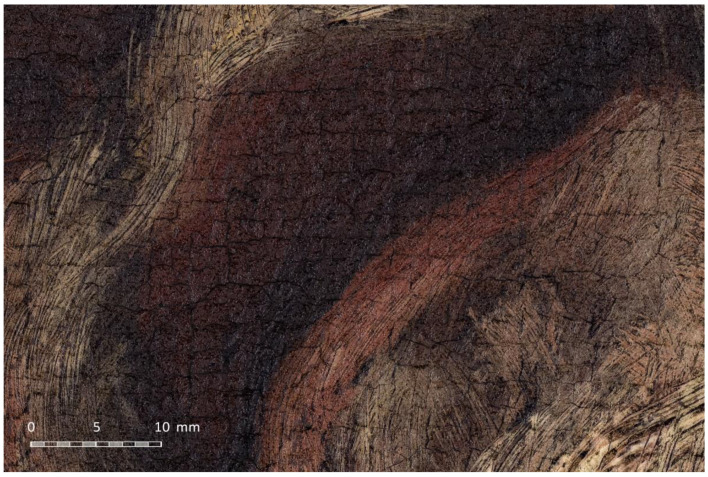
Fragment of the resulting gigapixel image.

As in the previous cases, the RAW developing process was carried out with RAWTherapee to apply the proper white balance and the specific colour profile to all the pictures at once. The stitching was carried out with PTGUI, which provided a gigapixel image (Figure 18b) with a total resolution of 30,884 × 44,228 px (1.36 gigapixels). In this case, the pixel density was 826 ppi, something lower than in the previous case due to the larger distance between the camera and the canvas. Figure 19 shows a cropped fragment of the gigapixel image focused on the saint’s mouth.

## 4. Discussion

The new proposed multi-viewpoint gigapixel capture techniques, compared to the conventional single-viewpoint capture technique, improve the image quality of the resulting gigapixel image. The parallel multi-viewpoint gigapixel capture technique is, in general, most recommended, so there is no loss of sharpness produced by narrow depth of field provided by telephoto lenses. The novel multi-viewpoint capture techniques imply using different shooting methods and moving the light system along with the camera to avoid changing specular reflections during the capture process.

Nevertheless, the single-viewpoint capture technique can be also a proper method when the depth of field provides an acceptable sharpness in the whole picture. The acceptable sharpness would depend on the focal lens, the diaphragm aperture value, the focus distance, and the camera sensor size. It can be calculated by using a free DOF calculator such as DOFMASTER [23]. 

From the experience acquired in this research, there are some practical considerations that can be very useful for future researchers, which will be discussed here. 

When documenting artworks with gigapixel resolution, the pixel density is an important aspect to bear in mind when planning the photographic session. A pixel density range between 600 ppi and about 1000 ppi was established to guarantee an excellent level of detail of the paintings. The maximum reachable pixel density value is always imposed by the photographic equipment, so it depends on the lens focal length, the camera distance, and the camera sensor resolution and size.

In order to maximize the pixel density, it would be advisable to use long focal lenses and to place the camera as close to the painting as possible (the minimum focusing distance of the lens can be the most limiting factor). 

The pixel density of the final gigapixel image can be calculated easily by Equation (1), which can be deduced geometrically:

(1) $$PD=\frac{S_{r}\;\cdot \;FL\;\cdot \;U_{f}}{D\;\cdot \;S_{w}}$$

where,

PD= Pixel Density of the Image (ppc or ppi) 

$S_{r}=$ Sensor Width Resolution (px)

FL= Lens Focal Length (mm)

$U_{f}=$ Unit Conversion Factor [use 10 for obtaining the pixel density in pixels per centimetre (ppc), and 25.4 for pixels per inch (ppi)]

D= Camera Distance (mm)

$S_{w}=$ Sensor Width (mm)

Obtaining a high pixel density is not necessarily a synonym of image quality, so a picture with a great pixel density can be totally blurred. For this reason, it is crucial to choose a lens providing excellent optical quality and to use it properly.

When using the proposed parallel multi-viewpoint technique, there is no problem with depth of field, so the camera sensor must remain parallel to the canvas always. The goal, in this case, is focusing the camera on the canvas as accurately as possible, maintaining the camera steady when shooting, and minimizing the loss of sharpness caused by the lens diffraction. 

In order to do so, it is necessary to use a tripod to maintain the camera totally stabilized. A measuring tape can be placed on the floor, parallel to the canvas, to work as a guide for the tripod legs so as to maintain the same distance from the camera to the canvas.

Additionally, it is advisable to focus and shoot the camera remotely to avoid small movements that would produce slightly blurred images. This can be done by using a computer or a mobile device with the software provided by the camera. The previous aspects are critical, especially when using long focal lenses in which any small movement would be extremely amplified on the camera sensor.

Moreover, setting the ISO setting to its minimum is mandatory to improve the image quality, as well as setting the proper exposure values to capture the entire dynamic range of the scene. In this sense, it is advisable to set the aperture to medium values, such as f8. Intermediate apertures would maximize the image sharpness, while avoiding diffraction problems. Once the aperture is set, the shutter speed can be determined accordingly to the diaphragm aperture to obtain the proper exposure. Visualizing the live histogram at this point can be very helpful to avoid clipping areas.

The image mosaic can be planned before starting the capture to know both the horizontal and the vertical camera displacements, as well as the total amount of shots that will be necessary to cover the entire painting. As mentioned before, a minimum overlap of about 30% between adjacent shots is advisable for a successful stitching. It is also recommended to shoot the camera in portrait mode to reduce the vertical camera movements, which are less convenient than the horizontal ones.

The camera vertical displacement can be determined by Equation (2):

(2) $$C_{v}=\frac{D\;\cdot \;S_{w}\;\cdot \;\left(100-P\right)}{100\;\cdot \;FL}$$

where,

$C_{v}=$ Camera Vertical Displacement (mm) 

D= Camera Distance (mm)

$S_{w}=$ Sensor Width (mm)

P= Image Overlap Percentage (%) (a minimum of 30% is advisable)

FL= Lens Focal Length (mm)

The camera horizontal displacement can be easily calculated as a fraction of the vertical displacement, so the sensor aspect ratio is known:

(3) $$C_{h}=\frac{C_{v}}{S_{a}}\;\;$$

where,

$C_{h}=$ Camera Horizontal Displacement (mm) 

$C_{v}=$ Camera Vertical Displacement (mm) 

$S_{a}=$ Sensor Aspect Ratio (normally 3:2 for professional cameras)

The total amount of shots composing the Photographic Mosaic (Rows and Columns) can be predicted as follows:

(4) $$R=\frac{H}{C_{v}}$$

(5) $$C=\frac{W}{C_{h}}$$

where,

R= Number of Rows of the Photographic Mosaic

C= Number of Columns of the Photographic Mosaic

H= Painting Height (mm)

W= Painting Width (mm)

$C_{h}=$ Camera Horizontal Displacement (mm) 

$C_{v}=$ Camera Vertical Displacement (mm)

A prediction of the resolution of the final gigapixel image in Gigapixels can be obtained from the next equation:

(6) $$RS=\frac{H\;\cdot \;W\;\cdot \;PD^{2}}{1\;\cdot \;10^{9}}$$

where,

RS= Total Resolution of the Gigapixel Image (Gp)

H= Painting Height (cm) 

W= Painting Width (cm)

PD= Pixel Density of the Image (ppc) 

As explained before, the resulting mosaic could be assembled with free stitching software, such as Hugin or with commercial one, such as PTGUI, which is very straightforward and fast. The resulting gigapixel image can be very heavy and difficult to handle unless a powerful computer is used. Fortunately, the dissemination of such heavy gigapixel images through the Internet can be carried out by decomposing the image in a multiresolution mosaic, which is also called pyramidal image. The process consists in applying an algorithm that generates different tiles from the gigapixel image, with different sizes and levels of resolution. Then, these image tiles can be loaded progressively in real time by using a specific html5 viewer. In this way, when the user demands more definition by zooming up, the viewer loads only the specific tiles for the visualization area.

When visualizing the resulting multiresolution mosaic through the Internet, there is no need to use powerful equipment, since even a conventional smartphone can manage this kind of pyramidal image. This technique is used in very well-known applications such as Google Maps or Google Earth.

It is important to mention that, due to the different kinds of display that the spectators can use for the visualization of the images online, it is advisable to generate the multiresolution mosaic using the standard colour profile sRGB, which is suitable for most of the devices. Nevertheless, the accuracy of the colour reproduction will depend on the gamut of the display and its proper calibration.

Zoomify [23] is one of the software packages that can provide multiresolution mosaics and has its own viewers, including a free version. There are another powerful opensource viewers, such as OpenSeadragon [24], which is the one used for this project.

**Figure 20:**
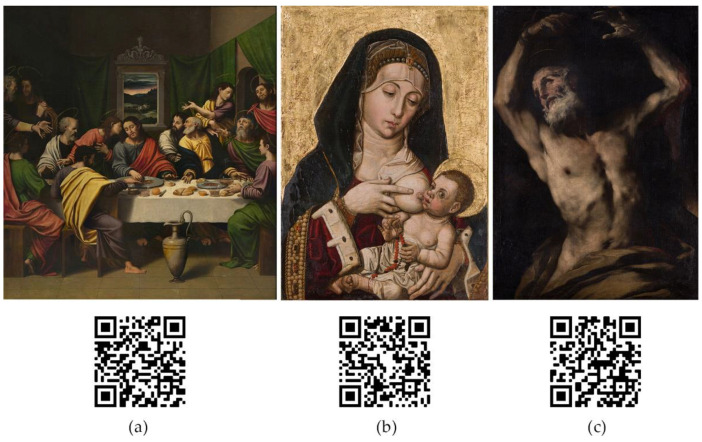
For an interactive gigapixel visualization of the documented artworks use the QR codes or follow the links below. (**a**) https://gpix.webs.upv.es/gpix/305.html (accessed on 19 August 2021); (**b**) https://gpix.webs.upv.es/gpix/279.html (accessed on 19 August 2021); (**c**) https://gpix.webs.upv.es/gpix/2197.html (accessed on 19 August 2021).

As a result, the gigapixel images that have been shown in this article can be visualized online by using the QR codes on Figure 20 or by following the links at the caption.

The rest of the museum imaging process can be solved using a proper web page design and this may depend on the needs of the researchers. In this case, Elementor [25], a simple and free web design plugin based on WordPress [26], was used to develop the entire webpage that allow the results to be disseminated: www.gpix.upv.es (accessed on 19 August 2021).
